# A possible primary cause of cancer: deficient cellular interactions in endocrine pancreas

**DOI:** 10.1186/1476-4598-11-63

**Published:** 2012-09-06

**Authors:** Maurice Israël

**Affiliations:** 1 Av. Aristide Briand 2, Bures sur Yvette, 91440, France

## Abstract

**Background:**

Cancer is a devastating type of disease. New and innovative ways to tackle cancers that have so far proved refractive to conventional therapies is urgently needed. It is becoming increasingly clear that, in addition to conventional therapeutics targeting by small molecules, that tumor cell metabolism presents new opportunities to target selectively specific cancer cell populations. Metabolic defects in cancer cells can be manifested in many ways that might not be readily apparent, such as altering epigenetic gene regulation for example.

The complex rewiring of metabolic pathways gives tumor cells a special advantage over differentiated cells, since they deplete body stores as fuel for their growth and proliferation. Tumor metabolism looks simpler when we consider that some enzymatic switches are in a neoglucogenic direction thereby depleting body stores. However, these pathways may be inadequately switched on by catabolic hormones (glucagon, epinephrine and cortisol) in a specific situation where anabolism is activated by, for example insulin released from beta pancreatic cells or IGF, inducing mitosis and synthesis that are powered by glucose catabolism. Such a hybrid metabolic situation would be reached if a pancreatic beta cell mechanism, mediated by GABA, failed to silence neighboring alpha cells and delta cells. The inhibitory transmitter GABA hyperpolarizes alpha and delta cells via their GABA A receptors, and blocks the release of glucagon and somatostatin. Alternatively, an anomaly of alpha cell channels, would lead to a similar situation. Whatever is the alteration, anabolism fails to silence catabolism and enzymatic switches controlled by kinases and phosphatases adopt an inadequate direction, leading to a hybrid metabolic rewiring found in cancer. It is daring to formulate such a hypothesis as this. However, it is quite possible that the starting point in cancer is an alteration of the endocrine pancreas, suppressing the mechanism by which beta cells silence the neighboring alpha and delta cells, with GABA and Zn^2+^.

## Introduction

In a recent review entitled: The metabolic advantage of tumor cells, published in Molecular Cancer [1] we analyzed a corpus of results on tumor cell metabolism describing a metabolic finality typical of cancer, in which body stores are depleted for building up the tumor mass. Tumor cells re-orient normal cellular processes; to create their metabolic advantage. We showed that signaling pathways, particularly the tyrosine kinase receptor cascade that is targeted by oncogenes, escapes from normal restrains. The role of a phosphatase targeted after methylation over the activated kinases was discussed. We described metabolic circuits operating in cancer, showing how some enzymes were switched on, while others were inhibited to create a flux of molecular building blocks beneficial to tumor cells. The proposed model indicates that some enzymatic switches adopt a neoglucogenic direction, while others promote oxidative glycolysis. This model takes into account the M2 Pyruvate kinase and PDH bottlenecks, the elevated citrate condensation and interruptions in Krebs and urea cycles. Observations such as the “Warburg effect” (increased lactate release), glutaminolysis, transaminations, poor arginine utilization, were all included in a coherent model, helping to explain the efficient metabolism of tumor cells [1]. In a more general context, it is probable that toxic chemicals or internal highly reactive products, such as superoxide are cell death factors and the replacement of terminally differentiated cells requires stem cells division. Mitotic cells may adopt metabolic features giving them a selective advantage over other cells that can be sustained by signaling network perturbations. The resulting metabolic changes are likely to induce epigenetic modifications that stabilize the metabolic advantage of these dividing cells; a pre-cancer situation then develops; however, this is in principle a reversible event. Then, inevitably, mutations select the most successful cells, progressing with the cells towards a cancerous phenotype. In this comment, we suggest that the complex metabolism of tumor cells was a possible consequence of an altered hormonal interaction, between neighboring pancreatic cells involving insulin and other factors released by beta cells [2].

This comment analyzes enzymatic switches regulated by insulin after a meal or after fasting and discusses their potential in cancer in particular. The channels sensing glucose; the beta cell interactions with neighboring alpha and delta cells; as well as more distant effects will be discussed. From this critique it can be inferred that a change in the physiology of pancreatic beta cells, failing to inhibit alpha and delta cells, is proposed to be a primary underlying cause for cancer leading to the metabolic rewiring described in the review “The metabolic advantage of tumor cells”.

### When blood glucose increases

**Figure 1:**
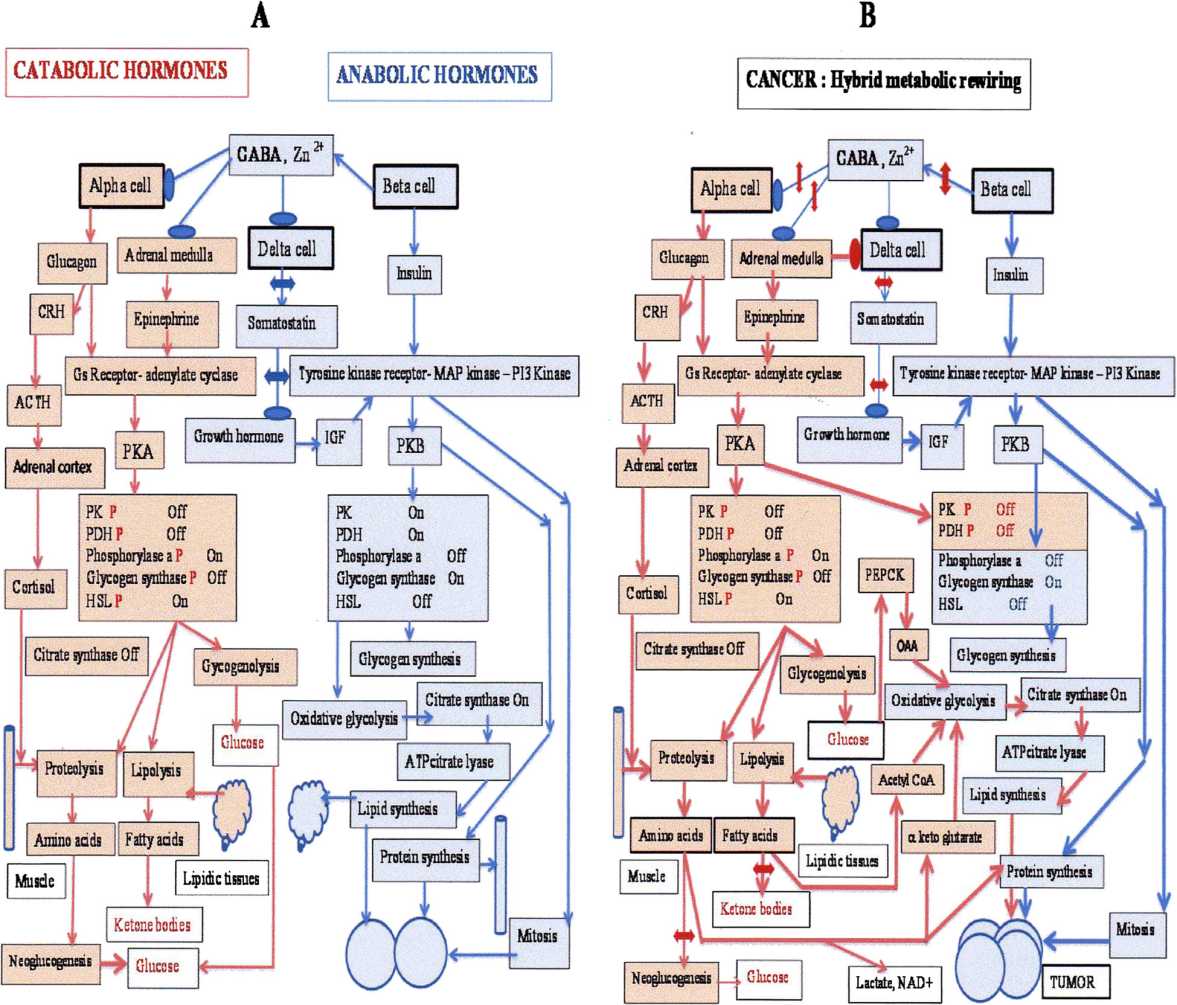
**Altered pancreatic beta cell mechanism silencing alpha and delta cells. A: right, anabolic hormones:** In hyperglycemia, beta cells release insulin; tyrosine kinase receptors elicit glucose uptake, synthetic processes, cells divide. Enzymes are dephosphorylated: PK, PDH, glycogensynthase are switched on, phosphorylase a, HSL are off; glycogen and lipids increase. These effects are induced via (PKB). Citratesynthase is active, controlled by NADH. Anabolic beta cells turn off with GABA- Zn^2+^ catabolic alpha cells. **A: left, catabolic hormones:** In hypoglycemia alpha cells release glucagon; adrenals release epinephrine. G_S_-coupled receptors activate adenylate cyclase, (PKA) phosphorylates enzymes: phosphorylase a, is switched on, hydrolyses glycogen; glycogensynthase is off. Neoglucogenesis is activated; (PK) and PDH blockade orient the pathway, sparing pyruvate for neoglucogenesis. Glucagon elicits (CRH) (ACTH) release triggering glucocorticoid-cortisol release from adrenals. Cortisol induces muscle proteolysis, providing amino acids for neoglucogenesis. Citratesynthase inhibition spares oxaloacetate for neoglucogenesis. Phosphorylated (HSL) provides fatty acids, giving ketone bodies. **B: Cancer hybrid metabolism:** GABA release interruption from beta cells, cancels alpha cells inhibition, eliciting a hybrid catabolism-anabolism **1-** In tumor cells, insulin induces mitosis and anabolism via PKB; citratesynthase is activate. But glucagon and epinephrine elicit, via PKA, phosphorylation and inhibition of PK and PDH. Low GABA increases epinephrine, inhibits somatostatin; reinforcing Growth Hormone-IGF- insulin actions. **2**- In stores, catabolic hormones trigger proteolysis and lipolysis. **3-** Metabolism is rewired below PK and PDH bottlenecks: tumor citratesynthase pulls the glucose flux; receiving oxaloacetate via (PEPCK), while acetylCoA comes from ketone bodies or fatty acids oxidized in peroxisomes (their mitochondrial transport is blocked by malonate forming tumor fatty acids via acetylCoA carboxylase). ATPcitratelyase receives the mitochondrial citrate efflux. Diverted from neoglucogenesis, amino acids form tumor proteins, feed lactatedehydrogenase via alanine transamination.

Hyperglycemia elicits the release of insulin from pancreatic beta cells causing increased cellular influx of glucose, thereby restoring the normal blood glucose level. Insulin like other anabolic hormones (Figure 1A right) acts on tyrosine kinase receptors inducing an anabolic process, synthesizing: principally glycogen, proteins, and lipids that are used as the building blocks of new cells. Oxidative glycolysis powers this anabolic process by providing the necessary energy. The insulin tyrosine kinase receptor activates complex downstream MAP and PI3 kinase routes, controlling both proliferation and metabolism. Lipogenesis and protein synthesis initiated by mTOR, are not represented, they are controlled by kinases and phosphatases exemplified by the glycogen synthesis model. The synthesis of glycogen for example, requires the activation of glycogen synthase by a phosphatase, dephosphorylating glycogen synthase. Conversely, the kinase GSK3 that blocks glycogen synthase is inactivated by insulin via PKB. Pyruvate kinase (PK) and pyruvate dehydrogenase (PDH) become active after being dephosphorylated, and these control the entry into the Krebs cycle. The first reaction of the cycle, the condensation of citrate synthase is boosted, while NADH reduces oxygen with electrons carried by the electron transport chain. As for the protons, they are pumped out the mitochondria inner membrane by the respiratory chain, and flow back through the F_1_-F_0_ ATPase to synthesize the necessary ATP. The beta cells are able to sense the level of glucose and release insulin; but they also will silence the neighboring delta cells that release somatostatin, an inhibitor on growth hormone and insulin like growth factor (IGF). By inhibiting delta cells, the beta cells reinforce the action of insulin mediated by IGF [3,4]. Above all, the messengers released by beta cells will also silence alpha cells releasing glucagon, whose actions are opposite to those of insulin [5].

### Effects of decreasing blood glucose

After starvation, hypoglycemia elicits the release of catabolic hormones: glucagon, epinephrine, cortisol that mobilize body stores for making nutrients: glucose and ketone bodies (Figure 1A left). The neoglucogenic route is fed by amino acids following the proteolysis of muscle protein that is controlled by glucocorticoids. Alanine coming from proteolysis is transaminated into pyruvate, which generates oxaloacetate (OAA) via pyruvate carboxylase. Following on, the enzyme phosphoenol pyruvate carboxykinase (PEPCK) converts OAA into phosphoenol pyruvate (PEP) building block of glucose. In this process, it is fundamental to block PK by phosphorylation; otherwise one would return from PEP to pyruvate. It is also necessary to block PDH by phosphorylation, favoring the pyruvate carboxylase reaction forming OAA, which starts the neoglucogenic pathway. Glucose can also come from the cleavage of glycogen, requiring the phosphorylation of phosphorylase b into active phosphorylase a, following the phosphorylation and activation of its specific kinase. Opposing this, the phosphorylation of glycogen synthase inactivates the enzyme blocking the synthesis of glycogen.

The synthesis of other cell nutrients such as ketone bodies requires the mobilization of lipid stores. A hormone sensitive lipase (HSL) [6] activated by phosphorylation hydrolyses triglycerides giving fatty acids forming through their beta oxidation acetyl CoA and ketone bodies.

These effects depend of protein kinase A (PKA) activated by glucagon and epinephrine, they are opposite to those of PKB stimulated by insulin signals.

The formation of glucose and ketone bodies would fail if liver citrate synthase at the entry of the Krebs cycle was not turned off. This occurs with a lower oxygen reduction increasing the NADH /NAD^+^ ratio, the oxidative mechanism is switched off, there is nothing to burn. In muscles, the Krebs cycle is not turned off, since they have to be active in stress conditions. With exercise, AMP increases and activates the Krebs cycle generating energy. In addition, AMP stimulates an AMP-dependent kinase, which inhibits acetyl CoA carboxylase, decreasing malonylCoA and the synthesis of fatty acids. The decrease of malonylCoA enhances the uptake of fatty acids in mitochondria and the production of ketone bodies.

In neoglucogenesis, there is a protection against muscle wasting, limiting the loss of muscle proteins and amino acids. When large amounts of amino radicals, coming from amino acids, enter in the urea cycle, arginine is formed. Arginine production elicits the secretion of growth hormone, which increases the incorporation of amino acids in muscles and protein synthesis, thereby cancelling the loss of amino acids induced by cortisol and catabolic hormones.

### In Cancer

In cancer, (Figure 1B) the enzymes PK, PDH, HSL, phosphorylase a, of body stores (Figure 1B left) are phosphorylated, via PKA activated by Gs receptors responding to catabolic hormones (glucagon, epinephrine). This situation mobilizes body stores as in starvation, compare to (Figure 1A left), which shows the effect of catabolic hormones. Tumor cells (Figure 1B right) display a hybrid situation, the enzymes PK, PDH are phosphorylated and blocked, via PKA and Gs receptors of tumor cells, as for catabolic hormone effects, while HSL, phosphorylase a, glycogen synthase are dephosphorylated, via PKB responding to tyrosine kinase receptors activated by insulin and IGF as in the case of anabolic hormone actions represented (Figure 1A right). In contrast to the “catabolic hormone situation” (Figure 1A left) tumor cells have an active citrate synthase, pulling the glycolytic flux in the glycolytic direction, to feed the condensation reaction of the Krebs cycle. How will tumor glycolysis reach the entry of the Krebs cycle and overcome the blockade of PK and PDH? This is not really a problem, since the enzyme PEPCK is fully reversible, and converts PEP, accumulated above the PK bottleneck, into OAA. The latter, is condensed with acetyl CoA coming from the mobilization of lipid stores (Figure 1B left and right). In tumors, the pyruvate resulting from alanine transamination gives lactate (Warburg effect) and NAD^+^ required for glycolysis to proceed at the glyceraldehydephosphate dehydrogenase step (Figure 1B left and right). It follows then that the supply of pyruvate to pyruvate carboxylase is interrupted. However, in tumors, there is a downstream blockade in the Krebs cycle, below the level of citrate synthase, favoring the efflux of citrate from mitochondria. This citrate is then cleaved by cytosolic ATP citratelyase, providing acetyl CoA for fatty acids and triglyceride synthesis, while the other enzyme product, OAA, drives the transaminases in the direction found in neoglucogenesis, which then consume amino acids and glutamine. In tumors these amino acids are diverted for making proteins rather than feeding the neoglucogenic pathway (Figure 1B left and right). As for the urea cycle it is active in tumors, as it is the case in neoglucogenesis, but the argininosuccinate synthase step is blocked.

The metabolic inference here is that the rewiring found in cancer consumes body stores, not for making glucose or ketone bodies, but for building the tumor while burning glucose (Figure 1B left and right).

### Anabolic hormones switch off catabolic hormones by a specific mechanism

#### Anabolic or catabolic situations

Figure 1A shows an overview of catabolic or anabolic hormonal effects on metabolism, where the selection between these processes is under the control of pancreatic beta cells, that hold the keys for switching on anabolism and switching off catabolism. Here, an interruption of the beta cell switch off system is represented, which predicts the metabolic changes observed in cancer (Figure 1B left and right). The metabolism of tumor cells has been described in detail [1]. The simplest summary, considers that part of the switches are in the neoglucogenic**-**ketogenic direction, depleting stores, as if the catabolic hormones: glucagon, epinephrine or cortisol where at work. While tumor cell switches responding to anabolic hormones: insulin and IGF, support anabolism, powered by oxidative glycolysis that is driven by an active citrate synthase condensation. However, even in tumor cells PK and PDH switches are off responding to catabolic hormones.

We do know that the tyrosine kinase receptor activated by insulin, stimulates oxidative glycolysis and triggers cell proliferation and anabolic processes. The cascades of signals that are activated are precisely targeted by many oncogenes. We also know that pancreatic beta cells that release insulin have to turn off the neighboring alpha cells releasing glucagon, with opposing actions taking place in starvation, when glucose is scarce. We should also bear in mind that glucagon, similarly to epinephrine, acts on G protein coupled receptors stimulating adenylate cyclase, the (Gs) type, increasing cAMP production. This in turn activates protein kinase A (PKA), which phosphorylates the set of enzymes (PK, PDH, HLP, phosphorylase a) mentioned above for making nutrients while mobilizing stores. The beta cell blocks these opposite glucagon and epinephrine effects [2]. The insulin beta cell granules release their contents in the extracellular space when beta cells are depolarized after sensing glycemia. Choline derivatives are also released; whose lipotropic action and role is discussed elsewhere [1][7]; they help the methylation of a phosphatase such as PP2A [8]. After methylation, it is targeted over specific sites, and counteracts the kinases activated by insulin tyrosine kinase signals. The insulin containing granules also contain other components such as Zn^2+^[9]. Moreover, beta cells are rich in GABA that is synthesized by the enzyme glutamate decarboxylase, which has been shown to be an autoantigen in insulin-dependent diabetes [10]. The release of GABA from beta cells has been well documented [11]. This transmitter acts on GABA A receptors found on the neighboring alpha and delta cells, triggering a chloride influx, which hyperpolarizes alpha cell and delta cells, blocking the release of glucagon and somatostatin [2,3]. The release of Zn^2+^ acts in synergy with GABA, blocking glucagon release from alpha cells [9]. At some distance, the GABA released with insulin, inhibits the release of epinephrine from adreno-medulla cells [12] that are activated by acetylcholine, but inhibited by GABA. In this way, the beta cell activates anabolism and oxidative glycolysis with insulin and inhibits with GABA the alpha cell, and the release of glucagon, switching off neoglucogenesis and lipolysis. The enzymes controlled by glucagon or epinephrine (PK, PDH, HLP phosphorylase a,) are no longer phosphorylated, since glucagon and epinephrine are no longer released, which keeps at rest the Gs coupled receptors, adenylate cyclase and PKA.

#### Glucose controls pancreatic cell ion channels and hormone release

Alpha, beta and delta pancreatic cells, sense the concentration of glucose via the levels of ATP in their cytosol; an increase of ATP closes membrane K^+^ channels that are inhibited by ATP (K _ATP_). This has the effect of depolarizing beta cells, whose electrical activity, depends greatly on L type calcium channels, and the influx of calcium elicits the release of insulin, GABA and other substances. These L type calcium channels are not inactivated by elevated depolarization, as a response to high glucose concentrations. The alpha cells that release glucagon are equipped with high affinity glucose transporters that start working at low glucose levels. The intracellular increase of ATP still has the effect of closing the K _ATP_ channels, but in alpha cells, the depolarization triggers the activity of voltage dependent sodium channels and calcium channels of the T and N types, which work at a low membrane depolarization, elicited by moderate changes in glucose levels. These channels are desensitized when the membrane depolarization is elevated, such as occurs if K _ATP_ channels are completely closed due to sensing of elevated glucose levels [13].The release of GABA from the beta cells inhibits the glucagon release from alpha cells that presumably occurs before they get self-inhibited at high glucose levels. As for the delta cells releasing somatostatin, the K _ATP_ channel sensors of glucose, elicits their depolarization. The resulting electrical activity depends of a variety of channels, including the L type calcium channel, as for beta cells, but also the P and Q types calcium channels that are activated at high-voltage. Release of GABA from beta cells inhibits somatostatin release from delta cells that are equipped with GABA A receptors [14]. If the release of GABA from beta cells decreases, glucagon release, and epinephrine release from adrenal medulla in turn increases, with the resulting epinephrine block of the delta cell somatostatin release [15].

It is probable that in resting conditions large amounts of GABA leak out from beta cells, keeping silent the alpha cells, hyperpolarized through their GABA A receptors. A decrease of glucose opens K _ATP_ channels, the beta cells become hyperpolarized, which decreases the resting efflux of GABA, the resultant depolarization of the alpha cell being greater than the hyperpolarization elicited by its own K _ATP_ channels. The depolarization of the alpha cell triggers their electrical activity and the calcium dependent release of glucagon correcting hypoglycemia. The situation is different in hyperglycemia; in the beta cell, hyperglycemia closes K _ATP_ channels, the depolarization eliciting the release of insulin, the release of GABA being essentially a resting release, nothing much changes. The alpha cells also sense hyperglycemia with K _ATP_ channels but in contrast to beta cells, their electrical activity depends on channels that are desensitized at elevated membrane depolarization, the alpha cell is electrically silent at high glucose, and glucagon is not released.

### Can tumor metabolism be fully explained?

The first possibility is that beta cells normally release insulin, but the release of GABA or Zn^2+^, is interrupted (as they are not necessarily simultaneous). Hence, insulin supports anabolism, while oxidative glycolysis driven by citrate synthase provides the energy for synthesizing new materials for dividing cells. The decreased release of GABA and Zn^2+^, will fail to block glucagon release from the alpha cells, while at some distance, the decrease of GABA enhances epinephrine release from adrenal cells. Glucagon and epinephrine act on Gs receptors, mediating PKA activation via adenylate cyclase and the phosphorylation of enzymes such as PK, PDH, HLP, phosphorylase a, that in a similar manner to starvation mobilizes body stores. But here, however, insulin has already activated synthetic processes that require energy and an active oxidative glycolysis, cells burn glucose while depleting stores! This situation is beneficial to the dividing cells that respond to tyrosine kinase signals. The result is that tumor cells mobilize body stores in anabolic and glycolytic conditions, to build the tumor mass using body stores, the metabolic rewiring linked to the blockade of PK and PDH in tumor cells and in body stores is schematized in Figure 1B, in tumor cells PK and PDH are inactivated via PKA signals, while the other tumor enzymes are boosted via PKB. Moreover, the release of glucagon acts on central neurons releasing corticotropin-releasing hormone (CRH), which increases ACTH release [16]. The latter, triggers the release of glucocorticoids (cortisol) from the adrenal cortex that binds to steroid receptors in muscles inducing proteolysis. Cortisol is a glucocorticoide and should support neoglucogenesis, but again, citrate synthase is abnormally active in tumor cells and pulls the flux in the glycolytic direction. The anti-inflammatory effect of glucocorticoid may also attenuate an immune reaction against nascent tumor cells. The result is a metabolism with a mix of neoglucogenic and glycolytic features, giving a selective advantage to tumor cells.

It is interesting to mention that PKA is involved in mRNA splicing mechanisms, controlling the selection of exons. In tumors, the embryonic form of pyruvate kinase, PK M2 with exon 10, replaces the adult enzyme PK M1 with exon 9 [17] and [18].

In summary, a decrease of GABA or Zn^2+^ contents and release from beta cells, would fail to inhibit glucagon and catabolic hormones. In addition, the central action of glucagon [16] increases growth hormone release and then IGF, which boosts the insulin effect. Somatostatin release from delta cells cannot attenuate Growth Hormone-IGF actions since the increase of epinephrine, resulting from the lower GABA released, blocks the delta cells [15], epinephrine instead does the job for GABA. In addition, a low choline release impairs the carboxy methylation of leucine 309 in the catalytic unit of PP2A phosphatase, thus the regulatory units are no longer recruited, removing the brake over activated kinases.

There is a second possibility for reaching a similar metabolic situation: an intrinsic alteration of alpha cell channels, which would keep them active at elevated glucose levels, when insulin is released from beta cells. The paracrine effects and more distant effects of GABA and Zn^2+^ and other substances that are released with insulin will still operate.

## Conclusion and inferences for future intervention strategies

In the scenario outlined herein, cancer may then result from a deficient composition of beta cell granules, or vesicles, or of GABA synthesized in their cytoplasm. The cocktail of factors released: low Zn^2+^, low GABA, normal insulin-corrects glycemia, but fails to inhibit the other pancreatic cells; the alpha cell, continues to release glucagon. In addition, at some distance, the adrenals will release more epinephrine. In the hypothalamus and hypophysis, glucagon increases CRH and ACTH, the latter triggers the release of cortisol from the adrenal cortex, cortisol mobilizes muscle protein stores, supporting the action of epinephrine and glucagon in conditions where insulin is secreted without its partners that block opposite actions.

Several experiments can be envisioned to test this provocative model. One may analyze the composition of beta cell granules and vesicles [19] and measure the GABA and glutamate decarboxylase. There are also histochemical procedures for Zn^2+^. The pharmacology of GABA receptors is well known; can we transform cells with compounds increasing glucagon release and catabolic hormones, in hyperglycemic conditions that activate insulin release? We may also manipulate the effects of catabolic hormones using known agonists, antagonists or toxins, changing cAMP and PKA actions, with the aim of such an experiment being to elicit a tumorigenic mechanism. Here, one would try to phosphorylate directly the enzymes mentioned, with compounds increasing cAMP, and then activate in a local population of cells, their insulin tyrosine kinase receptor signaling, their oxidative metabolism and citrate synthase. Whether these cells become tumor cells depleting body stores for their benefit is a key question. One may also study the effects of bicuculine, an antagonist of GABA A receptors, in hyperglycemic conditions that elicit an insulin release. The PP2A brake over signaling kinases might also be removed using choline uptake inhibitors, anti folates, as this should inhibit the carboxy methylation of PP2A. We have in a previous review [1] listed compounds acting on downstream targets associated to cancer, citrate [20] hydroxycitrate, lipoid acid, that gave interesting results [21], a useful low arginine diet [22] and other targets were discussed. In this comment, we have possibly identified an upstream target that leads to cancer. If further experiments demonstrated that this is indeed the case, the starting point of cancer would be a specific alteration of Langerhans cells in the endocrine pancreas; it is not insulin that is decreased but its partners, GABA, Zn^2+^ and choline, creating conditions that appear inevitably to lead to tumor formation.

Finally, it may be interesting to increase the GABA content of beta cells, possibly by transfecting them with a plasmid encoding glutamate decarboxylase, since this autoantigen gives a type 1 diabetes. A stimulation of glutamate decarboxylase with vitamin B_6_ also deserves investigation. Some time ago we described a chemiluminescent GABA assay that would be useful for monitoring this transmitter [23]. As for Zn^2+^, it deserves to be studied in this context, as well as the glutamatergic transmission in the pancreas. Phosphatases are particularly interesting, choline, B_12_, folate, would support a protective methylation of PP2A, as discussed in other works. At the very least, this comment suggests new approaches and possible experiments in the field of cancer development and treatment.

## Abbreviations

PK, Pyruvate kinase; PDH, Pyruvate dehydrogenase; HLP, Hormone sensitive lipase; PEPCK, Phosphoenol pyruvate carboxykinase; IGF, Insulin like growth factor; PEP, Phosphoenol pyruvate; OAA, Oxaloacetate; GABA, Gamma amino butyric acid; K ATP, K + channels inhibited by ATP; Gs, G protein coupled receptor stimulating adenylate cyclase; PKA, Protein kinase A; PKB, Protein kinase B; GSK3, Glycogen synthase kinase 3; CRH, Corticotropin-releasing hormone; ACTH, Adrenocorticotropic hormone.

## Competing interests

The author declares that he has no competing interests.

## Author contributions

MI is a neurochemist, retired CNRS Director, MD, PHD

Av. Aristide Briand 2, Bures sur Yvette 91440 France.

mauisrael@wanadoo.fr
